# Structural, Electronic, and Thermodynamic Properties of Tetragonal *t*-Si*_x_*Ge_3−*x*_N_4_

**DOI:** 10.3390/ma11030397

**Published:** 2018-03-07

**Authors:** Chenxi Han, Changchun Chai, Qingyang Fan, Jionghao Yang, Yintang Yang

**Affiliations:** 1 Key Laboratory of Ministry of Education for Wide Band-Gap Semiconductor Materials and Devices, School of Microelectronics, Xidian University, Xi’an 710071, China; hancxxd@163.com (C.H.); ccchai@mail.xidian.edu.cn (C.C.); ytyang@xidian.edu.cn (Y.Y.); 2 Xi’an Institute of Applied Optics, Xi’an 710065, China; jhyang1988@163.com

**Keywords:** Si*_x_*Ge_3−*x*_N_4_, mechanical properties, electronic properties, thermodynamic properties

## Abstract

The structural, mechanical, anisotropic, electronic, and thermal properties of *t*-Si_3_N_4_, *t*-Si_2_GeN_4_, *t*-SiGe_2_N_4_, and *t*-Ge_3_N_4_ in the tetragonal phase are systematically investigated in the present work. The mechanical stability is proved by the elastic constants of *t*-Si_3_N_4_, *t*-Si_2_GeN_4_, *t*-SiGe_2_N_4_, and *t*-Ge_3_N_4_. Moreover, they all demonstrate brittleness, because *B/G* < 1.75, and *v* < 0.26. The elastic anisotropy of *t*-Si_3_N_4_, *t*-Si_2_GeN_4_, *t*-SiGe_2_N_4_, and *t*-Ge_3_N_4_ is characterized by Poisson’s ratio, Young’s modulus, the percentage of elastic anisotropy for bulk modulus *A_B_*, the percentage of elastic anisotropy for shear modulus *A_G_*, and the universal anisotropic index *A*^U^. The electronic structures of *t*-Si_3_N_4_, *t*-Si_2_GeN_4_, *t*-SiGe_2_N_4_, and *t*-Ge_3_N_4_ are all wide band gap semiconductor materials, with band gaps of 4.26 eV, 3.94 eV, 3.83 eV, and 3.25 eV, respectively, when using the Heyd-Scuseria-Ernzerhof (HSE06) hybrid functional. Moreover, *t*-Ge_3_N_4_ is a quasi-direct gap semiconductor material. The thermodynamic properties of *t*-Si_3_N_4_, *t*-Si_2_GeN_4_, *t*-SiGe_2_N_4_, and *t*-Ge_3_N_4_ are investigated utilizing the quasi-harmonic Debye model. The effects of temperature and pressure on the thermal expansion coefficient, heat capacity, Debye temperature, and Grüneisen parameters are discussed in detail.

## 1. Introduction

IV A Group nitrides have attracted considerable interest as high-performance ceramics due to their outstanding physical properties [1]. Silicon nitride has many advantages, such as high strength, wear resistance, a high decomposition temperature, oxidation resistance, outstanding thermal shock resistance, a low friction coefficient, and low corrosion resistance, thus making it the ideal material for use in engineering ceramics [2]. Germanium nitride also has advantages, such as corrosion resistance and an adjustable band gap [3,4]. 

Si_3_N_4_ under normal temperature and pressure has two polymorphs: *α*-Si_3_N_4_ and *β*-Si_3_N_4_. It is generally accepted that *α*-Si_3_N_4_ is a metastable state, and *β*-Si_3_N_4_ is the low-temperature phase of Si_3_N_4_ crystal [5]. In 1999, Zerr et al. synthesized the third polymorph, *γ*-Si_3_N_4_ (cubic spinel structure, also named *c*-Si_3_N_4_ [6]) [7]. Since then, more researchers have studied the transitions among the three phases of Si_3_N_4_. Togo et al. [8] found that the phase transition pressure of *β* → *γ* was 12.5 GPa at 300 K. Through X-ray diffraction experiments, Kruger et al. [9] found that the *α*-Si_3_N_4_ could remain stable at room temperature and in the pressure range of 0–48 GPa. The phase transition pressure of *α* → *γ* was 15.22 GPa at 300 K, as reported by Yu and Chen [6]; they also found that the stability order of the three types of polymorphs was *β*-Si_3_N_4_ > *α*-Si_3_N_4_ > *γ*-Si_3_N_4_. Kroll predicted two new phases of Si_3_N_4_: post-spinel and *wII*- [10,11]. Yu et al. [12] investigated the structural and elastic properties of post-spinel and *wII*- of Si_3_N_4_. They found that post-spinel and *wII*-Si_3_N_4_ were stable at 160 GPa and 0 GPa, respectively. The post-spinel Si_3_N_4_ could be obtained when *γ*-Si_3_N_4_ was at 152.5 GPa and 0 K, while *β* → *wII* occurred at 20 GPa and 300 K.

Ge_3_N_4_ was first identified in 1930, when *β*-Ge_3_N_4_ was first synthesized [13]. Ruddlesden and Popper [14] found that the *α* phase was closely related to *β*-Ge_3_N_4_; however, it is generally believed to be a metastable phase under ambient conditions. Ge_3_N_4_ is considered to be a better candidate for obtaining the *wII* phase than Si_3_N_4_, because the transition to higher coordination states could be easier; it also may occur at lower pressures in germanium nitride than in silicon nitride because of the larger ionic size of Ge [15]. McMillan et al. [16] observed the first-order phase transition between the *β* and *σ* (space group: *P*3) phases at 23 GPa and 298 K. They also predicted the *β* → *P*$\bar{6}$ → *σ* phase transitions at 20 GPa and 28 GPa, respectively. Wang et al. [17] found that *γ*-Ge_3_N_4_ could retain its stability up to 69.2 GPa at room temperature. The critical pressure of the *β* → *wII* phase transition was 10.7 GPa (at 300 K), and the *β* → *wII* transformation occurred at 13.5 GPa and 1100 K; further compression led to the *wII* → *γ* transition at 35.7 GPa, as reported by Luo et al. [18] In addition to silicon nitride and germanium nitride, IV A Group and V A Group elements compounds have been investigated, such as Si_3_P_4_ and Ge_3_P_4_ [19,20,21], W_3_N_4_ [22,23,24], BN [25,26,27,28,29,30] and C_3_N_4_ [31,32,33,34,35].

Recently, Cui et al. discovered three new phases of Si_3_N_4_: the tetragonal phase *t*-Si_3_N_4_, the monoclinic phase *m*-Si_3_N_4_, and the orthorhombic phase *o*-Si_3_N_4_ [36]. They found that Δ*H* for *m*-Si_3_N_4_ and *t*-Si_3_N_4_ were smaller than *γ*-Si_3_N_4_ below 2.9 GPa and 2.5 GPa, respectively. *o*-Si_3_N_4_ is a high pressure phase, with P_t_ = 198 GPa from *γ*-Si_3_N_4_. Subsequently, other researchers studied the physical properties of *t*-Si_3_N_4_, *m*-Si_3_N_4_, and *o*-Si_3_N_4_ [37,38,39]. Yao and Chen reported on the structural properties, mechanical properties, Vickers hardnesses, and electronic properties of *t*-Ge_3_N_4_, *m*-Ge_3_N_4_, and *o*-Ge_3_N_4_ [37]. When the pressure was below 20 GPa, the formation enthalpies of *t*-Ge_3_N_4_, *m*-Ge_3_N_4_, and *o*-Ge_3_N_4_ were, negative indicating they are stable. Fan et al. [38] investigated the elastic anisotropic and electronic properties of *m*-Si_3_N_4_, *o*-Si_3_N_4_, and *t*-Si_3_N_4_ under high pressure. They found that the *m*-Si_3_N_4_ transition from the direct band gap to the indirect band gap state occurred at ~32 GPa. Most of the Si_3_N_4_ and Ge_3_N_4_ semiconductors are wide band gap semiconductors; however, most of them are also indirect band gap semiconductors, such as *α*-Si_3_N_4_, *β*-Si_3_N_4_, *o*-Si_3_N_4_, *t*-Si_3_N_4_, post-spinel Si_3_N_4_, and *α*-Ge_3_N_4_ [7,36,40,41]. It is reported that the band gaps of (Si_1−*x*_Ge*_x_*)_3_N_4_ could be adjusted by the ratio of Si:Ge [42]. The cubic spinel *c*-Si_3_N_4_, *c*-SiGe_2_N_4_, and *c*-Ge_3_N_4_ were theoretically predicted to have wide and direct band gaps of 3.45 eV, 1.85 eV, and 2.22 eV, respectively, and *c*-GeSi_2_N_4_ had an indirect band gap of 2.56 eV and a direct band gap of 2.64 eV [43]. The cubic spinel SiGe_2_N_4_ has been shown to be of particular interest, because it is a stable compound with a direct band gap [44]. Ma et al. [45] studied the structural, mechanical, elastic, anisotropic, and electronic properties of the monoclinic phase of *m*-Si*_x_*Ge_3−*x*_N_4_ (*x* = 0, 1, 2, 3) alloys. Their results indicated that the *m*-Si*_x_*Ge_3−*x*_N_4_ (*x* = 0, 1, 2, 3) alloys are all direct and wide band gap semiconductor materials, and that the band gaps could be adjusted from 3.34–5.08 eV. From Ref. [43,44,45], it could be envisioned that the proper mixing of (Si, Ge)_3_N_4_ in the cubic spinel and monoclinic phases may lead to compounds with appropriate properties, which we needed. For example, the band gaps of (Si, Ge)_3_N_4_ could be tuned by changing the Si:Ge ratio. These tunable band gap materials are particularly useful in applications such as light-emitting diodes (LEDs), hybrid solar cells, sensors, and photocatalysts.

Since there are no studies regarding the mixing of (Si, Ge)_3_N_4_ in the tetragonal phase, we propose two new double nitrides, *t*-Si_2_GeN_4_ and *t*-SiGe_2_N_4_, in the *I*-42*m* space group. In the present work, we investigate and discuss the structural, elastic, electronic, and thermodynamic properties for *t*-Si*_x_*Ge_3−*x*_N_4_ (*x* = 0, 1, 2, 3) alloys, which would be helpful for future experiments and theoretical explorations.

## 2. Calculation Methods

This work was performed based on the density functional theory (DFT) [46,47] using the Cambridge Serial Total Energy Package (CASTEP) plane-wave code [48]. The calculations were performed with the generalized gradient approximation (GGA) in the form of the Perdew–Burke–Ernzerhof (PBE) functional, [49], the Perdew–Burke–Ernzerhof functional for solids (PBEsol) [50], and the local density approximation (LDA) in the form of Ceperley and Alder data, as parameterized by the Perdew and Zunger (CA–PZ) [46] exchange correlation potential. The valence electron configurations of Si, Ge, and N atoms were Si-3*s*^2^3*p*^2^, Ge-4*s*^2^4*p*^2^, and N-2*s*^2^2*p*^3^, respectively. The cut-off energy was set as 500 eV. The Brillouin zone of *t*-Si_3_N_4_, *t*-Si_2_GeN_4_, *t*-SiGe_2_N_4_, and *t*-Ge_3_N_4_ could be described by the *k*-points of 10 × 10 × 5, 10 × 10 × 5, 9 × 9 × 5, and 9 × 9 × 5 using the Monkhorst–Pack mesh, [51] respectively. The crystal structures were optimized by the Broyden–Fletcher–Goldfarb–Shanno (BFGS) algorithm [52]. The self-consistent convergence of the total energy was 5 × 10^−6^ eV/atom; the maximum force on the atom was 0.01 eV/Å, the maximum ionic displacement was within 5 × 10^−4^ Å, and the maximum stress was within 0.02 GPa. The thermodynamic properties of *t*-Si*_x_*Ge_3−*x*_N_4_ (*x* = 0, 1, 2, 3) alloys were investigated utilizing the quasi-harmonic Debye model [53,54].

## 3. Results and Discussion

### 3.1. Structural Properties

**Figure 1:**
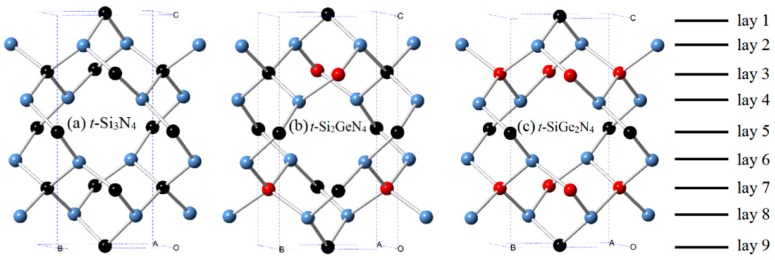
Crystal structures of *t*-Si_3_N_4_ (**a**); *t*-Si_2_GeN_4_ (**b**); and *t*-SiGe_2_N_4_ (**c**) at ambient pressure. The silicon, germanium, and nitrogen atoms are represented as black, red, and blue spheres, respectively.

**Figure 2:**
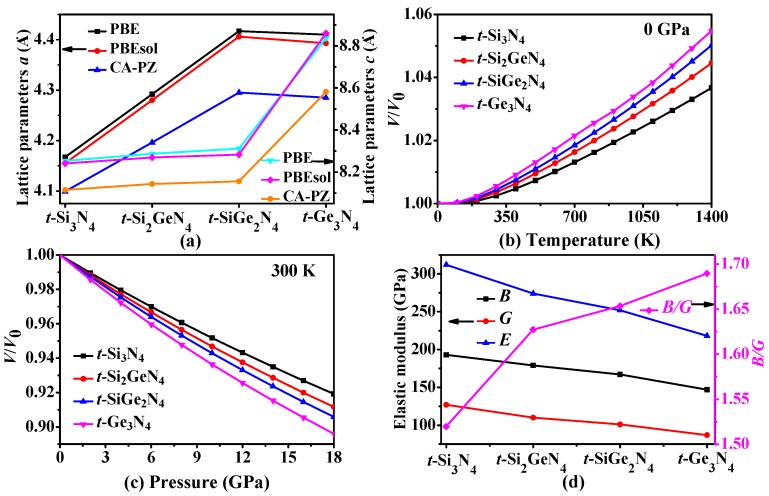
Calculated lattice parameters *a* and *c* (**a**) of *t*-Si_3_N_4_, *t*-Si_2_GeN_4_, *t*-SiGe_2_N_4_, and *t*-Ge_3_N_4_ by PBE, PBEsol, and CA–PZ methods; the volume *V*/*V*_0_ compression as functions of temperature (**b**) and pressure (**c**); the elastic moduli for *t*-Si_3_N_4_, *t*-Si_2_GeN_4_, *t*-SiGe_2_N_4_, and *t*-Ge_3_N_4_ (**d**).

The crystal structures of *t*-Si_3_N_4_, *t*-Si_2_GeN_4_, *t*-SiGe_2_N_4_, and *t*-Ge_3_N_4_ are displayed in Figure 1. The *t*-Si_2_GeN_4_, *t*-SiGe_2_N_4_, and *t*-Ge_3_N_4_ are obtained when germanium atoms replace the Si atom of *t*-Si_3_N_4_ with the smallest energy. The equilibrium lattice parameters of *t*-Si*_x_*Ge_3−*x*_N_4_ and *c*-Si*_x_*Ge_3−*x*_N_4_ (*x* = 0, 1, 2, 3) alloys with the related reference data are listed in Table 1. As seen, our calculated lattice parameters for *t*-Si_3_N_4_ and *c*-Si*_x_*Ge_3−*x*_N_4_ (*x* = 0, 1, 2, 3) alloys are in excellent agreement with the existing results. The lattice parameters of *t*-Si*_x_*Ge_3−*x*_N_4_ (*x* = 0, 1, 2, 3) alloys as a function with the Ge component are shown in Figure 2a. From Figure 2a, the lattice parameter *a* increases with an increase in the percentage of the germanium composition, except for *t*-Ge_3_N_4_. The lattice parameter *a* of *t*-Ge_3_N_4_ is slightly smaller than *t*-SiGe_2_N_4_; the possible cause of this phenomenon is that the angle of ∠Si-N-Ge (113.95°) and ∠Ge-N-Ge (112.16°) in *t*-SiGe_2_N_4_ decreases in *t*-Ge_3_N_4_ (∠Si-N-Ge: 112.86°; ∠Ge-N-Ge: 111.75°) parallel to the *ac* plane. Moreover, the lattice parameter *c* of *t*-Si_3_N_4_, *t*-Si_2_GeN_4_, and *t*-SiGe_2_N_4_ is almost the same; however, the lattice parameter *c* of *t*-Ge_3_N_4_ is far greater than that of the others. The bond length of the silicon nitrogen bond is certainly smaller than that of the germanium nitrogen bond in the same structure. From *t*-Si_3_N_4_ and *t*-Ge_3_N_4_, as the substitution of germanium atoms for silicon atoms increases and the substitution occurs primarily along the *c* axis, the growth of germanium nitrogen bonds is mainly manifested in the *c* axis direction. In Figure 1c, the silicon or germanium atoms occupy five of the nine layers of atoms along the *c* axis. From *t*-Si_3_N_4_ to *t*-Si_2_GeN_4_, the germanium atoms only replace two of the silicon atoms; thus, at this time, the increase along the *c* axis is very small. In addition, from *t*-Si_2_GeN_4_ to *t*-SiGe_2_N_4_, the germanium atoms are replaced on the same layers, so the increase along the *c* axis is very small. From *t*-SiGe_2_N_4_ to *t*-Ge_3_N_4_, all of the germanium atoms replace the silicon atoms, so the lattice parameter *c* suddenly increases.

In addition, we further investigate the influence of pressure and temperature for *t*-Si_3_N_4_, *t*-Si_2_GeN_4_, *t*-SiGe_2_N_4_, and *t*-Ge_3_N_4_. The ratio of volume *V/V*_0_ affected by temperature and pressure is shown in Figure 2b,c. As shown in Figure 2b,c, the volume of *t*-Si_3_N_4_, *t*-Si_2_GeN_4_, *t*-SiGe_2_N_4_, and *t*-Ge_3_N_4_ increases as the temperature increases. When *T* < 300 K, the growth rate is very small; however, when *T* > 300 K, the volume is found to increase linearly. Furthermore, the ratio of *V/V*_0_ clearly decreases as the pressure increases, approaching a linear decrease. Moreover, Figure 2b,c indicates that the effect of the pressure on the *V/V*_0_ is more significant than that of the temperature in the pressure and temperature ranges that are considered in our study. In addition, the volume of *t*-Ge_3_N_4_ changes more than do those of *t*-Si_3_N_4_, *t*-Si_2_GeN_4_, and *t*-SiGe_2_N_4_.

### 3.2. Mechanical and Anisotropic Properties

**Figure 3:**
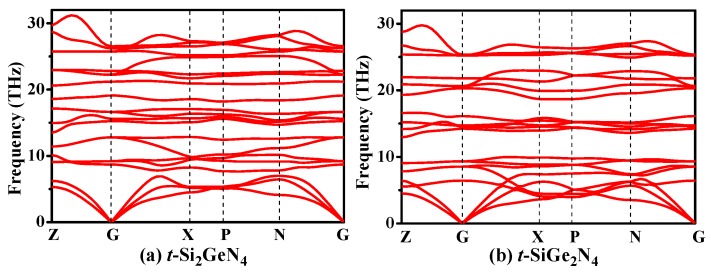
Phonon spectra for *t*-Si_2_GeN_4_ (**a**) and *t*-SiGe_2_N_4_ (**b**).

The elastic constants, elastic moduli, and Poisson’s ratio for *t*-Si*_x_*Ge_3−*x*_N_4_, *c*-Si*_x_*Ge_3−*x*_N_4_, and *m*-Si*_x_*Ge_3−*x*_N_4_ (*x* = 0, 1, 2, 3) alloys are all listed in Table 2. The elastic constant is an index that reflects the ability of materials to resist elastic deformation. From Table 2, the elastic constants of the *t*-Si_2_GeN_4_ and *t*-SiGe_2_N_4_ satisfy Born’s mechanical stability criteria of tetragonal symmetry [57]; as a result, *t*-Si*_x_*Ge_3−*x*_N_4_ (*x* = 0, 1, 2, 3) alloys are mechanically stable. In order to confirm the stability of *t*-Si_2_GeN_4_ and *t*-SiGe_2_N_4_, the phonon spectra are calculated at ambient pressure, which are shown in Figure 3. There is no imaginary frequency, proving that the alloys are stable.

The Voigt–Reuss–Hill approximation [60] is typically used to calculate bulk modulus *B*_H_ and shear modulus *G*_H_. The Young’s modulus *E* and Poisson’s ratio *v* could be obtained as: *E* = 9*B*_H_*G*_H_/(3*B*_H_ + *G*_H_) and *v* = (3*B*_H_ − 2*G*_H_)/[2(3*B*_H_ + *G*_H_)], respectively. The bulk modulus *B*_H_ represents the resistance to compressibility, and the shear modulus *G*_H_ represents the resistance to plastic deformation. The calculated bulk modulus *B*_H_ and shear modulus *G*_H_ are listed in Table 2. For *t*-Si*_x_*Ge_3−*x*_N_4_ (*x* = 0, 1, 2, 3) alloys, the bulk modulus are larger than the shear modulus, which indicates that it is more difficult for volume deformation to occur in alloys than shape deformation. The bulk modulus *B*_H_, shear modulus *G*_H_, and Young’s modulus *E* all decrease with an increase in the Ge component. Comparing *t*-Si*_x_*Ge_3−*x*_N_4_, *c*-Si*_x_*Ge_3−*x*_N_4_, and *m*-Si*_x_*Ge_3−*x*_N_4_ (*x* = 0, 1, 2, 3) alloys, the elastic modulus for *t*-Si*_x_*Ge_3−*x*_N_4_ (*x* = 0, 1, 2, 3) alloys is slightly larger than that for *m*-Si*_x_*Ge_3−*x*_N_4_ (*x* = 0, 1, 2, 3) alloys, whereas it is much smaller than that for *c*-Si*_x_*Ge_3−*x*_N_4_ (*x* = 0, 1, 2, 3) alloys.

Pugh [61] proposed that the ratio of *B*_H_*/G*_H_ could accurately distinguish the brittleness or ductility of a material. If *B*_H_*/G*_H_ > 1.75, then the material exhibits a ductile property; otherwise, it exhibits a brittle property. The ratio *B*_H_*/G*_H_ of *t*-Si*_x_*Ge_3−*x*_N_4_ (*x* = 0, 1, 2, 3) alloys as a function with a Ge component are shown in Figure 2d. From Figure 2d, although the value of *B*_H_*/G*_H_ increases with the increasing percentage of the Ge composition, they are all less than 1.75. This suggests that *t*-Si*_x_*Ge_3−*x*_N_4_ (*x* = 0, 1, 2, 3) alloys all exhibit brittleness. Poisson’s ratio *v* can also be used to distinguish the brittleness or ductility of a material [62]. If *v* >0.26, then the material will behave in a ductile manner; otherwise, the material demonstrates brittleness. The calculated Poisson’s ratios *v* of *t*-Si*_x_*Ge_3−*x*_N_4_, *c*-Si*_x_*Ge_3−*x*_N_4_ and *m*-Si*_x_*Ge_3−*x*_N_4_ (*x* = 0, 1, 2, 3) alloys are all listed in Table 2. From Table 2, all of the *v* are less than 0.26. Thus, *t*-Si*_x_*Ge_3−*x*_N_4_ (*x* = 0, 1, 2, 3) alloys all demonstrate brittleness; this is consistent with the conclusion based on *B*_H_*/G*_H_*.* Moreover, *c*-Si*_x_*Ge_3−*x*_N_4_ (*x* = 0, 1, 2, 3) alloys are quite brittle because of the small ratio of *B*_H_*/G*_H_; *m*-SiGe_2_N_4_ is ductile; and *m*-Si_3_N_4_, *m*-Si_2_GeN_4_, and *m*-Ge_3_N_4_ are brittle in nature.

**Figure 4:**
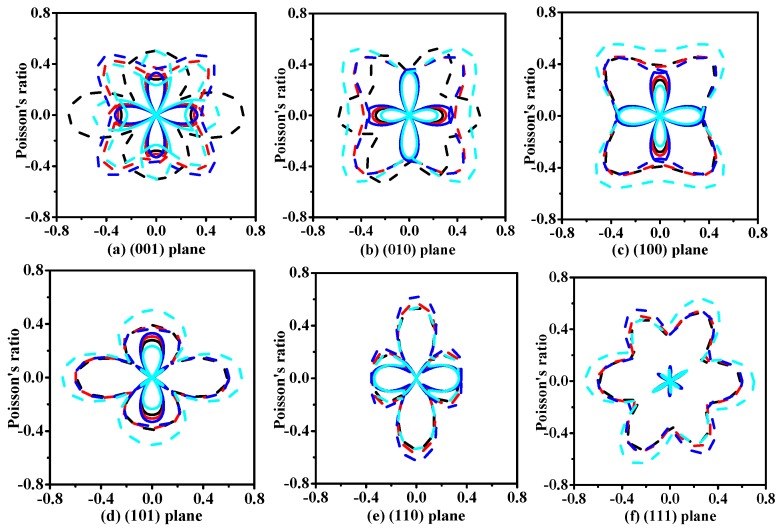
Two-dimensional (2D) representations of Poisson’s ratio for *t*-Si*_x_*Ge_3−*x*_N_4_ (*x* = 0, 1, 2, 3) alloys in the (001) plane (**a**); (010) plane (**b**); (100) plane (**c**); (101) plane (**d**); (110) plane (**e**); and (111) plane (**f**). The dash dot and solid lines represent the maximum and minimum values, respectively. The black, red, blue, and cyan lines represent the Poisson’s ratios of *t*-Si_3_N_4_, *t*-Si_2_GeN_4_, *t*-SiGe_2_N_4_, and *t*-Ge_3_N_4_, respectively.

It is well known that elastic anisotropy has important implications in engineering science and crystal physics. The directional dependence of the anisotropy is calculated by the elastic anisotropy measures (ELAM) [63,64] code. The two-dimensional (2D) representation of Poisson’s ratio *v* in (001), (010), (100), (101), (110), and (111) planes for *t*-Si*_x_*Ge_3−*x*_N_4_ (*x* = 0, 1, 2, 3) alloys are shown in Figure 4. Clearly, the 2D figures of Poisson’s ratio all have a great deviation degree from a circular shape, indicating that the *t*-Si*_x_*Ge_3−*x*_N_4_ (*x* = 0, 1, 2, 3) alloys exhibit anisotropy in Poisson’s ratio. Moreover, the four alloys present the different degree of anisotropy on Poisson’s ratio in different planes. The maximum Poisson’s ratio for *t*-Si_3_N_4_, *t*-Si_2_GeN_4_, *t*-SiGe_2_N_4_, and *t*-Ge_3_N_4_ are 0.59, 0.60, 0.62, and 0.70, respectively, following the sequence *t*-Si_3_N_4_ > *t*-Si_2_GeN_4_ > *t*-SiGe_2_N_4_ > *t*-Ge_3_N_4_. The following directional dependence of anisotropy is usually described by vector direction (*θ*, *φ*), where *θ* (*φ*) represents the angle between the vector and the *x*-axis (*z*-axis) positive direction, and they are expressed in radians [63,64]. The positions of the maximum Poisson’s ratio for *t*-Si_3_N_4_, *t*-Si_2_GeN_4_, *t*-SiGe_2_N_4_, and *t*-Ge_3_N_4_ appear at *θ* = 0.79, *φ* = 0.00; *θ* = 2.36, *φ* = 0.00; *θ* = 1.57, *φ* = 3.93; and *θ* = 2.33, *φ* = 0.00, respectively.

**Figure 5:**
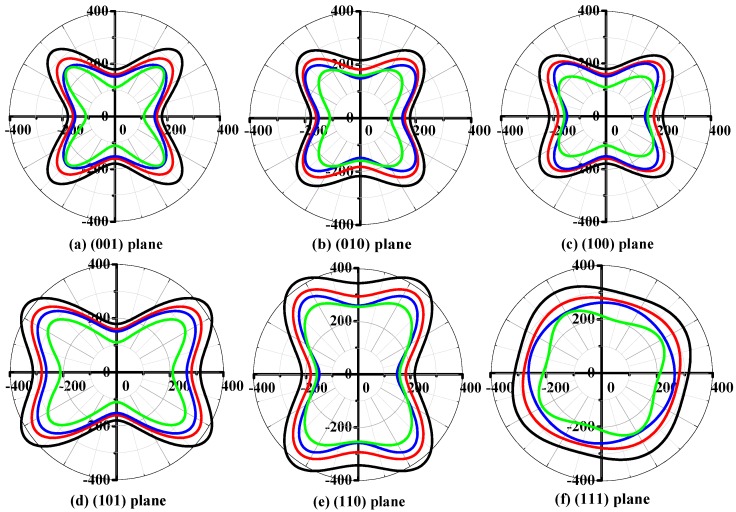
2D representation of Young’s modulus for *t*-Si*_x_*Ge_3−*x*_N_4_ (*x* = 0, 1, 2, 3) alloys in the (001) plane (**a**); (010) plane (**b**); (100) plane (**c**); (101) plane (**d**); (110) plane (**e**); and (111) plane (**f**). The black, red, blue, and green lines represent the Poisson’s ratios of *t*-Si_3_N_4_, *t*-Si_2_GeN_4_, *t*-SiGe_2_N_4_, and *t*-Ge_3_N_4_, respectively. All units are in GPa.

The 2D figures of Young’s modulus for *t*-Si_3_N_4_, *t*-Si_2_GeN_4_, *t*-SiGe_2_N_4_, and *t*-Ge_3_N_4_ are shown in Figure 5. Clearly, *t*-Si*_x_*Ge_3−*x*_N_4_ (*x* = 0, 1, 2, 3) alloys exhibit large anisotropy in Young’s modulus in all of the planes except the (111) plane. The Young’s modulus of the materials in the (111) plane are approximately circular in shape, indicating that the materials exhibited the smallest anisotropy in the (111) plane. In addition, the shapes of the four lines are similar; thus, the anisotropy of Young’s modulus should change little with an increasing percentage of Ge composition. The maximum (minimum) values of *t*-Si_3_N_4_, *t*-Si_2_GeN_4_, *t*-SiGe_2_N_4_, and *t*-Ge_3_N_4_ are 421 GPa (179 GPa), 373 GPa (159 GPa), 347 GPa (149 GPa), and 306 GPa (111 GPa), respectively. The positions of the maximum values of *t*-Si_3_N_4_, *t*-Si_2_GeN_4_, *t*-SiGe_2_N_4_, and *t*-Ge_3_N_4_ are *θ* = 2.19, *φ* = 3.93; *θ* = 0.95, *φ* = 3.93; *θ* = 2.19, *φ* = 3.93; and *θ* = 2.17, *φ* = 3.93, respectively. The minimum value of *t*-Si_3_N_4_, *t*-Si_2_GeN_4_, and *t*-Ge_3_N_4_ appears at *θ* = 1.57, *φ* = 0.00, and the minimum value of *t*-SiGe_2_N_4_ appears at *θ* = 0.00, *φ* = 0.00. The values of *E*_max_/*E*_min_ for *t*-Si_3_N_4_, *t*-Si_2_GeN_4_, *t*-SiGe_2_N_4_, and *t*-Ge_3_N_4_ are 2.352, 2.346, 2.329, and 2.757, respectively. From these results, *t*-Ge_3_N_4_ has the largest anisotropy, and those of *t*-Si_3_N_4_, *t*-Si_2_GeN_4_, and *t*-SiGe_2_N_4_ are similar.

Moreover, the elastic anisotropic of the material could be shown by the percentage of elastic anisotropy for the bulk modulus *A*_B_, the percentage of elastic anisotropy for the shear modulus *A*_G_, and the universal anisotropic index *A*^U^: *A*^U^ = 5*G*_V_/*G*_R_ + *B*_V_/*B*_R_ − 6, *A*_B_ = (*B*_V_ − *B*_R_)/(*B*_V_ + *B*_R_), and *A*_G_ = (*G*_V_ − *G*_R_)/(*G*_V_ + *G*_R_) [65]. The calculated values of *A*_B_, *A*_G_ and *A*^U^ of *t*-Si*_x_*Ge_3−*x*_N_4_, *c*-Si*_x_*Ge_3−*x*_N_4_, and *m*-Si*_x_*Ge_3−*x*_N_4_ (*x* = 0, 1, 2, 3) alloys are all listed in Table 3. From Table 3, *t*-Si*_x_*Ge_3−*x*_N_4_ (*x* = 0, 1, 2, 3) alloys show a slight degree in anisotropy in the bulk modulus, because *A*_B_ is very close to zero. For *t*-Si*_x_*Ge_3−*x*_N_4_ (*x* = 0, 1, 2, 3) alloys, the percentage of elastic anisotropy for shear modulus *A*_G_ is the smallest for *t*-Si_2_GeN_4_, and is slightly smaller than that of *t*-Si_3_N_4_; meanwhile, *t*-Ge_3_N_4_ has the largest *A*_G_. For *t*-Si_3_N_4_, *c*-Si_3_N_4_, and *m*-Si_3_N_4_, the *A*_G_ of *c*-Si_3_N_4_ is approximately half that of *t*-Si_3_N_4_. The *t*-Si*_x_*Ge_3−*x*_N_4_ (*x* = 0, 1, 2, 3) alloys exhibit the largest anisotropy compared with *c*-Si*_x_*Ge_3−*x*_N_4_ and *m*-Si*_x_*Ge_3−*x*_N_4_ (*x* = 0, 1, 2, 3) alloys, except for *m*-SiGe_2_N_4_. At the same time, *m*-SiGe_2_N_4_ exhibits the largest *A*_G_ among the *t*-Si*_x_*Ge_3−*x*_N_4_ (*x* = 0, 1, 2, 3) alloys, *c*-Si*_x_*Ge_3−*x*_N_4_ (*x* = 0, 1, 2, 3) alloys, and *m*-Si*_x_*Ge_3−*x*_N_4_ (*x* = 0, 1, 2, 3) alloys. The greater the value of *A*^U^, the greater the anisotropy. The values of the universal anisotropic index *A*^U^ of the *t*-Si*_x_*Ge_3−*x*_N_4_ (*x* = 0, 1, 2, 3) alloys are 1.231, 1.261, 1.283, and 1.637, respectively. It was also found that *t*-Ge_3_N_4_ has the greatest *A*^U^, indicating that *t*-Ge_3_N_4_ has the largest anisotropy among *t*-Si*_x_*Ge_3−*x*_N_4_ (*x* = 0, 1, 2, 3) alloys, which is consistent with the results of *A*_B_ and *A*_G_.

### 3.3. Electronic Properties

**Figure 6:**
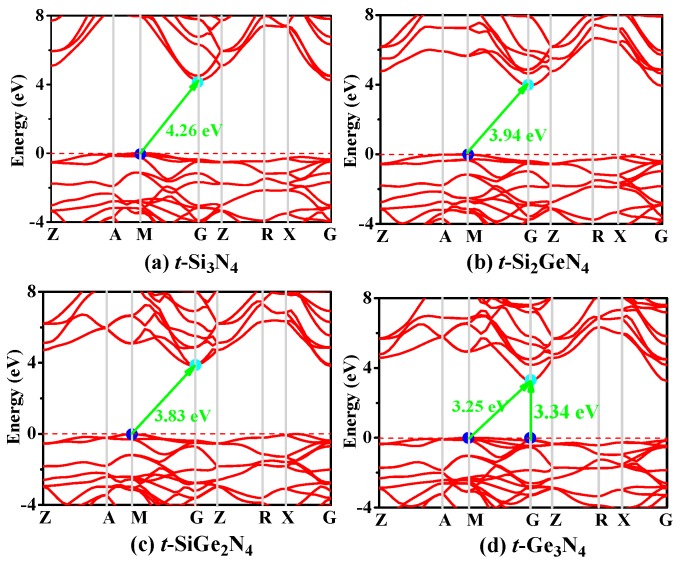
Electronic band structures of *t*-Si_3_N_4_ (**a**); *t*-Si_2_GeN_4_ (**b**); *t*-SiGe_2_N_4_ (**c**); and *t*-Ge_3_N_4_ (**d**) with the HSE06 hybrid functional.

As is known, the electronic structure determines the physical and chemical properties of materials. The electronic band structures of *t*-Si_3_N_4_, *t*-Si_2_GeN_4_, *t*-SiGe_2_N_4_, and *t*-Ge_3_N_4_ are shown in Figure 6, according to calculations with the HSE06 hybrid functional [66,67]. From Figure 6, the *t*-Si*_x_*Ge_3−*x*_N_4_ (*x* = 1, 2, 3) alloys are all indirect band gap semiconductor materials, and *t*-Ge_3_N_4_ is a quasi-direct band gap semiconductor material. In addition, the band gaps of *t*-Si_3_N_4_, *t*-Si_2_GeN_4_, *t*-SiGe_2_N_4_, and *t*-Ge_3_N_4_ are 4.26 eV, 3.94 eV, 3.83 eV, and 3.25 eV, respectively; the conduction band minima are all located at the M point, and the valence band maxima are all located at the G point. With the increasing percentage of the Ge composition, the band gap decreases. For *t*-Ge_3_N_4_, the direct gap at G point is 3.34 eV, which is slightly larger than the indirect gap of 3.25 eV; as a result, *t*-Ge_3_N_4_ is a quasi-direct gap semiconductor. The calculated band gaps that utilize other functions, such as PBE, PBEsol, and CA–PZ, are listed in Table 1. It is known that the calculated band gaps are usually underestimated with DFT; i.e., the true band gap is larger than the calculated results. The band gaps of *t*-Si_3_N_4_, *t*-Si_2_GeN_4_, *t*-SiGe_2_N_4_, and *t*-Ge_3_N_4_ with the HSE06 hybrid functional are found to be much larger than those calculated by other functions.

**Figure 7:**
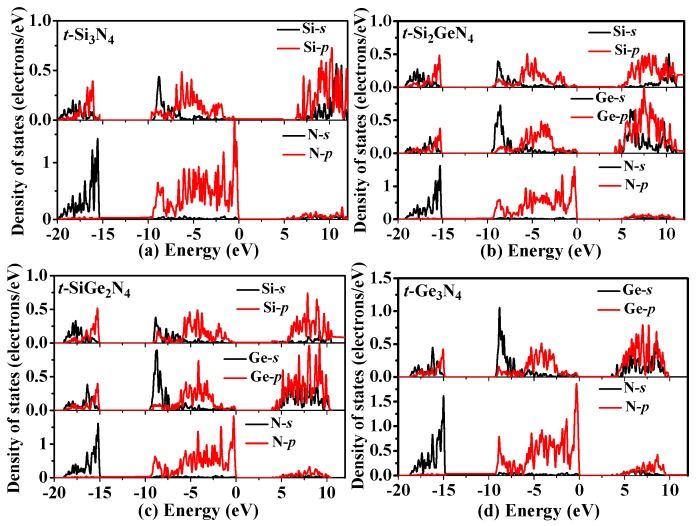
Comparison of total density of states and partial density of states for *t*-Si_3_N_4_ (**a**); *t*-Si_2_GeN_4_ (**b**); *t*-SiGe_2_N_4_ (**c**); and *t*-Ge_3_N_4_ (**d**). The black and red curves represent the *s* states and *p* states, respectively, of silicon, germanium, and nitrogen atoms.

To further investigate the nature of the electronic band structure for *t*-Si_3_N_4_, *t*-Si_2_GeN_4_, *t*-SiGe_2_N_4_, and *t*-Ge_3_N_4_, we also investigated the partial density of states (PDOS) for *t*-Si*_x_*Ge_3−*x*_N_4_ (*x* = 0, 1, 2, 3) alloys displayed in Figure 7. Since the *t*-Si*_x_*Ge_3−*x*_N_4_ (*x* = 0, 1, 2, 3) alloys are all tetragonal crystal and Si and Ge belong to the IV A Group, their PDOS are similar and consist of three regions. The first region is from approximately −20 eV to −15 eV; the second and third regions are from −10 eV to the Fermi energy (*E*_F_) and from 5 eV to 12 eV, respectively. For *t*-Si_3_N_4_ and *t*-Ge_3_N_4_, the first region originates from the N-*s*, Si-*s/p* or Ge-*s/p* states. The second region is from the N-*p* states, with a mixture of Si-*s/p* or Ge-*s/p* states. The last region is primarily from Si/Ge-*p* states and a mixture of Si/Ge-*s* states and N-*p* states. For *t*-Si_2_GeN_4_ and *t*-SiGe_2_N_4_, the first region is primarily from N-*s* states, with an admixture from Si-*s/p* and Ge-*s/p* states. The second region primarily originates from N-*p* states, with significant contributions from the Si-s and Ge-*s* states between −9 and −7 eV, and the Si-*p* and Ge-*p* states between −7 eV and *E*_F_. The Ge-*s*/*p* and Si-*p* states dominate the last region, ranging from 5 eV to approximately 10 eV.

### 3.4. Thermodynamic Properties

**Figure 8:**
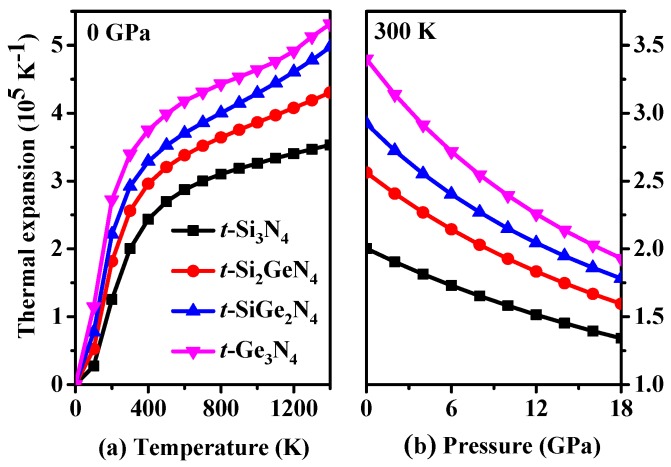
Temperature (**a**) and pressure (**b**) dependence of the thermal expansion coefficients for *t*-Si_3_N_4_, *t*-Si_2_GeN_4_, *t*-SiGe_2_N_4_, and *t*-Ge_3_N_4_.

The thermodynamic properties of semiconductors are very important at higher temperatures and pressures. In this work, the highest temperature is 1400 K, and the highest pressure is 18 GPa. In the above conditions, the thermal expansion coefficient *α*, the heat capacities *C*_V_ and *C*_P_, and the Debye temperature *Θ_D_* are all investigated here. The thermal expansion coefficient describes how the size of a material changes with a change in temperature. Specifically, the thermal expansion coefficient measures the fractional change in size per degree change in temperature at a constant pressure; it is one of the important parameters to measure for determining the thermodynamic properties of materials. The values of the thermal expansion coefficient *α* of *t*-Si*_x_*Ge_3−*x*_N_4_ (*x* = 0, 1, 2, 3) alloys as functions of temperature and pressure are shown in Figure 8. Figure 8a shows that *α* increases exponentially with an increase in temperature until 300 K at 0 GPa. When *T* > 300 K, the growth rate of *α* decreases, and *α* increases linearly after *T* > 800 K. The growth rate of thermal expansion *α* at a high temperature is far less than that at a low temperature; i.e., the temperature dependence of *α* is very small at a high temperature. In addition, at a given temperature, *α* decreases with an increase in pressure, and the decline rate decreases with the increase in pressure. The effect of the temperature on the thermal expansion coefficient *α* is found to be more significant than that of pressure. Note that the thermal expansion coefficient *α* is the smallest for *t*-Si_3_N_4_, whereas *t*-Ge_3_N_4_ has the largest thermal expansion coefficient *α* in *t*-Si*_x_*Ge_3−*x*_N_4_ (*x* = 0, 1, 2, 3) alloys.

**Figure 9:**
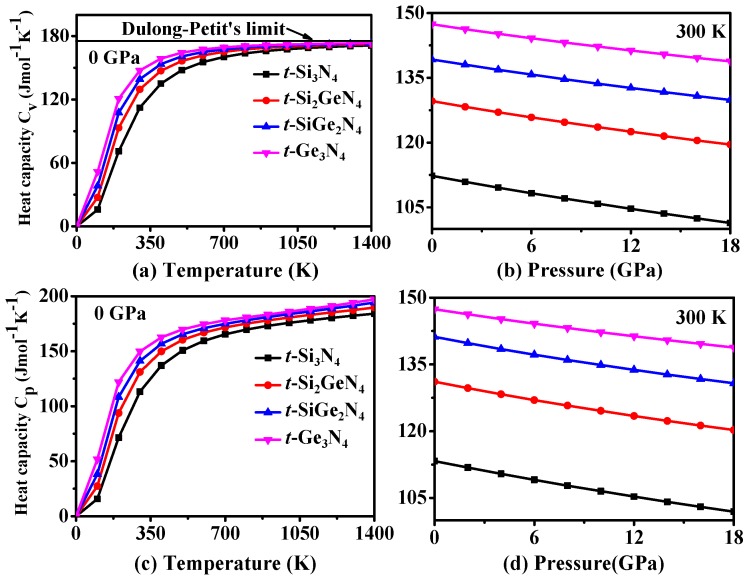
Calculated specific volume *C*_V_ as a function of temperature (**a**) and as a function of pressure (**b**); the pressure heat capacity *C*_P_ as a function of temperature (**c**) and as a function of temperature pressure (**d**) for *t*-Si_3_N_4_, *t*-Si_2_GeN_4_, *t*-SiGe_2_N_4_, and *t*-Ge_3_N_4_.

The variation of the heat capacity (*C*_V_) at constant volume and the heat capacity (*C*_p_) of *t*-Si_3_N_4_, *t*-Si_2_GeN_4_, *t*-SiGe_2_N_4_, and *t*-Ge_3_N_4_ at a constant pressure versus temperature and pressure variations are shown in Figure 9. The curves of heat capacity for *t*-Si_3_N_4_, *t*-Si_2_GeN_4_, *t*-SiGe_2_N_4_, and *t*-Ge_3_N_4_ have similar tendencies, as do the variations of the temperature and pressure that are shown in Figure 9. From Figure 9a, the heat capacity curves are proportional to *T*^3^ when *T* < 300 K, and the growth rate of the heat capacity decreases above 300 K. For *T* > 800 K, the heat capacity gradually approaches the fixed value of the Dulong–Petit limit (174.54 J mol^−1^ K^−1^) [68]. As shown in Figure 9c, the change regulation of *C*_P_ is similar to that of *C*_V_ below 800 K. *C*_P_ increases linearly as the temperature increases above 800 K. From Figure 9b,d, the effect of pressure and temperature on heat capacity is the opposite; i.e., the heat capacities for *t*-Si*_x_*Ge_3−*x*_N_4_ (*x* = 0, 1, 2, 3) alloys decrease with an increase in pressure at a given temperature. Moreover, the sensitivity of heat capacity to temperature is far greater than that to pressure. Figure 9 also shows that the heat capacity for *t*-Si*_x_*Ge_3−*x*_N_4_ (*x* = 0, 1, 2, 3) alloys follows the sequence: *t*-Ge_3_N_4_ > *t*-SiGe_2_N_4_ > *t*-Si_2_GeN_4_ > *t*-Si_3_N_4_.

**Figure 10:**
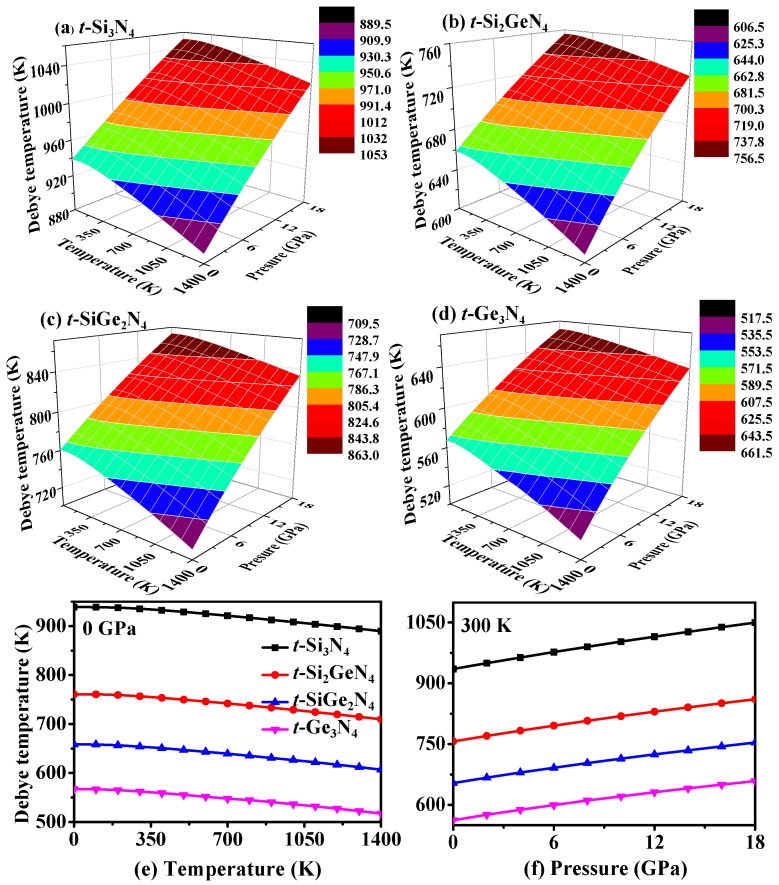
(**a**–**d**) Three-dimensional contour plots of the Debye temperature versus pressure and temperature for *t*-Si_3_N_4_ (**a**); *t*-Si_2_GeN_4_ (**b**); *t*-SiGe_2_N_4_ (**c**); and *t*-Ge_3_N_4_ (**d**). The Debye temperature as functions of temperature (**e**) and pressure (**f**) for *t*-Si_3_N_4_, *t*-Si_2_GeN_4_, *t*-SiGe_2_N_4_, and *t*-Ge_3_N_4_.

Figure 10a–d shows the variations of Debye temperature *Θ_D_* of *t*-Si*_x_*Ge_3−*x*_N_4_ (*x* = 0, 1, 2, 3) alloys with the temperature and pressure. From Figure 10a–d, the change regulation of *Θ_D_* for *t*-Si*_x_*Ge_3−*x*_N_4_ (*x* = 0, 1, 2, 3) alloys has the same trend under variable temperature and variable pressure. In addition, the effect of the pressure on *Θ_D_* is found to be more significant than that of temperature. To better illustrate the effect of temperature and pressure variation on the *Θ_D_* of *t*-Si*_x_*Ge_3−*x*_N_4_ (*x* = 0, 1, 2, 3) alloys, the *Θ_D_* values as functions of temperature or pressure are shown in Figure 10e–f. From Figure 10e, the *Θ_D_* remains nearly constant from 0 K to 200 K, and when *T* > 200 K, the *Θ_D_* decreases linearly. Moreover, at a given temperature, the value of *Θ_D_* almost increases monotonously with an increase in pressure, as shown in Figure 10f. From 0 K to 1400 K, *Θ_D_* decreases by 5.2%, 6.7%, 7.8%, and 8.8% for *t*-Si_3_N_4_, *t*-Si_2_GeN_4_, *t*-SiGe_2_N_4_, and *t*-Ge_3_N_4_, respectively, and from 0 GPa to 18 GPa, *Θ_D_* decreases by 12.2%, 13.7%, 15.3%, and 17.1%. In addition, the larger the Ge composition is, the larger the influence to *Θ_D_* is. The values of *Θ_D_* for *t*-Si_3_N_4_, *t*-Si_2_GeN_4_, *t*-SiGe_2_N_4_, and *t*-Ge_3_N_4_ calculated by the quasi-harmonic Debye model (938 K, 761 K, 658 K, and 567 K) are in agreement with those calculated by the elastic modulus [65] (*Θ*_D_ = (*h*/*k*_B_)[(3*n*/4π)(*N*_A_*ρ*/*M*)]^1/3^*v_m_*, *v_m_* = [(2/$v_{s}^{3}$ + 1/$v_{p}^{3}$)/3]^−1/3^, *v_p_* = [(*B* + 4*G*/3)/*ρ*]^1/2^, *v_s_* = (*G*/*ρ*)^1/2^; 937 K, 767 K, 666 K, and 571 K) at 0 K and 0 GPa.

## 4. Conclusions

In the present work, the structural, mechanical, elastic anisotropic, electronic, and thermal properties of *t*-Si_3_N_4_, *t*-Si_2_GeN_4_, *t*-SiGe_2_N_4_, and *t*-Ge_3_N_4_ in the tetragonal phase were investigated using density functional theory. The mechanically stable forms of *t*-Si_2_GeN_4_ and *t*-SiGe_2_N_4_ were proved by elastic constants. The *t*-Si*_x_*Ge_3−*x*_N_4_ (*x* = 0, 1, 2, 3) alloys were all found to be brittle according to Poisson’s ratio *v* and *B*/*G*. The elastic modulus was found to decrease with an increase in the proportion of Ge. Also, *t*-Ge_3_N_4_ was found to exhibit the largest anisotropy among the *t*-Si*_x_*Ge_3−*x*_N_4_ (*x* = 0, 1, 2, 3) alloys. It was found that *t*-Si_3_N_4_, *t*-Si_2_GeN_4_, and *t*-SiGe_2_N_4_ were indirect band gap semiconductors, but *t*-Ge_3_N_4_ was a quasi-direct gap semiconductor material. The band gaps of *t*-Si*_x_*Ge_3−*x*_N_4_ (*x* = 0, 1, 2, 3) alloys decreased with germanium content, which was suitable for visible light applications such as LEDs and photocatalysts. Moreover, all of the alloys considered were found to be wide band gap semiconductor materials, which indicated that transistors made from them could withstand higher temperatures and voltages, and the switching speed would be faster. In addition, their thermodynamic properties were investigated in detail utilizing the quasi-harmonic Debye model. The thermal expansion coefficient *α* and heat capacity were found to be more susceptible to temperature than pressure, whereas the Debye temperature was found to be more susceptible to pressure than temperature. These results would provide reference data for the experiments and make current theoretical research on these alloys more plenitude. It also could be envisioned that, by adjusting the Si:Ge ratio, the double nitrides in the tetragonal phases would lead to alloys with tailored electronic and thermodynamic properties for specific applications.

## Figures and Tables

**Table 1:** The lattice parameters *a* and *c* (in Å) and band gaps *E*g (in eV) of *t*-Si_3_N_4_, *t*-Si_2_GeN_4_, *t*-SiGe_2_N_4_, and *t*-Ge_3_N_4_ and *c*-Si_3_N_4_, *c*-Si_2_GeN_4_, *c*-SiGe_2_N_4_, and *c*-Ge_3_N_4._ PBE: Perdew–Burke–Ernzerhof functional; PBEsol: Perdew–Burke–Ernzerhof functional for solids; CA–PZ: Perdew and Zunger.

Materials	PBE			PBEsol			CA–PZ		
	*a*	*c*	*E*g	*a*	*c*	*E*g	*a*	*c*	*E*g
*t*-Si_3_N_4_	4.167	8.253	3.05	4.155	8.241	2.89	4.099	8.115	2.99
-	4.131 ^1^	8.168	3.15	-	-	-	-	-	-
-	4.166 ^2^	8.249	-	-	-	-	-	-	-
*t*-Si_2_GeN_4_	4.292	8.287	2.74	4.280	8.269	2.60	4.196	8.143	2.71
*t*-SiGe_2_N_4_	4.417	8.312	2.31	4.406	8.283	2.33	4.295	8.156	2.59
*t*-Ge_3_N_4_	4.410	8.838	1.79	4.393	8.86	1.80	4.285	8.582	2.67
*c*-Si_3_N_4_	7.763	-	-	7.751	-	-	7.639	-	-
-	7.773 ^3^	-	-		-	-	-	-	-
-	7.770 ^4^	-	-		-	-	-	-	-
*c*-Si_2_GeN_4_	7.934	-	-	7.918	-	-	7.767	-	-
-	8.001 ^5^	-	-		-	-	-	-	-
*c*-SiGe_2_N_4_	8.111	-	-	8.095	-	-	7.909	-	-
-	8.182 ^6^	-	-		-	-	-	-	-
*c*-Ge_3_N_4_	8.296	-	-	8.289	-	-	8.040	-	-
-	8.288 ^7^	-	-		-	-	-	-	-
-	8.300 ^8^	-	-		-	-	-	-	-

^1^ Ref. [36]. ^2^ Ref. [39]. ^3^ Ref. [12]. ^4^ Ref. [55]-experimental. ^5^ Ref. [43]. ^6^ Ref. [44]. ^7^ Ref. [18]. ^8^ Ref. [56]-experimental.

**Table 2:** Calculated elastic constants *C*_ij_ (in GPa), bulk modulus *B*_H_, shear modulus *G*_H_, and Young’s modulus *E* (in GPa), the ratio of *B*_H_/*G*_H_ and Poisson’s ratio *v* of *t*-Si*_x_*Ge_3−*x*_N_4_, *c*-Si*_x_*Ge_3−*x*_N_4_, and *m*-Si*_x_*Ge_3−*x*_N_4_ (*x* = 0, 1, 2, 3) alloys compared with other calculated results.

Materials	*C*_11_	*C*_12_	*C*_13_	*C*_33_	*C*_44_	*C*_66_	*B*_H_	*G*_H_	*E*	*B*_H_/*G*_H_	*v*
*t*-Si_3_N_4_	277	148	144	314	176	206	193	127	312	1.52	0.230
-	277 ^1^	152	145	312	178	207	194	126	311	1.54	-
*t*-Si_2_GeN_4_	254	138	137	278	158	171	179	110	274	1.63	0.245
*t*-SiGe_2_N_4_	241	127	131	243	150	151	167	101	252	1.65	0.248
*t*-Ge_3_N_4_	200	126	110	233	127	148	147	87	218	1.69	0.253
*c*-Si_3_N_4_	524	177	-	-	333	-	293	256	595	1.14	0.161
-	512 ^2^	177	-	-	331	-	289	-	-	-	-
-	-	-	-	-	-	-	290 ^7^	-	-	-	-
*c*-Si_2_GeN_4_	453	167	-	-	298	-	263	222	520	1.18	0.170
-			-	-		-	258 ^8^	-	-	-	-
*c*-SiGe_2_N_4_	440	151	-	-	251	-	247	201	474	1.229	0.179
-	441 ^3^	150	-	-	129	-	247	-	-	-	-
-	494 ^4^	172	-	-	288	-	283	-	-	-	-
*c*-Ge_3_N_4_	375	140	-	-	223	-	218	172	409	1.27	0.187
-	368 ^5^	145	-	-	223	-	220	-	-	-	-
-	-	-	-	-	-	-	296 ^9^	-	-	-	-
*m*-Si_3_N_4_^6^	257	36	131	353	93	92	165	110	270	1.51	0.228
*m*-Si_2_GeN_4_^6^	242	37	126	316	76	85	154	97	241	1.59	0.239
*m*-SiGe_2_N_4_^6^	235	42	164	363	60	42	170	78	202	2.20	0.303
*m*-Ge_3_N_4_^6^	180	34	110	274	57	61	126	73	184	1.73	0.258

^1^ Ref. [39]. ^2^ Ref. [12]. ^3^ Ref. [44]. ^4^ Ref. [58]. ^5^ Ref. [18]. ^6^ Ref. [45]. ^7^ Ref. [55], ^8^ Ref. [43], ^9^ Ref. [59]-experimental.

**Table 3:** Calculated bulk modulus *B*_V_, *B*_R_ and shear modulus *G*_V_, *G*_R_ by the Voigt and Reuss method respectively; percent compressibility of bulk modulus and shear modulus factors (*A*_B_, *A*_G_) % and universal anisotropic indexes *A*^U^ for *t*-Si*_x_*Ge_3−*x*_N_4_, *c*-Si*_x_*Ge_3−*x*_N_4_, and *m*-Si*_x_*Ge_3−*x*_N_4_ (*x* = 0, 1, 2, 3) alloys.

Materials	*B*_V_	*B*_R_	*A*_B_	*G*_V_	*G*_R_	*A*_G_	*A*^U^
*t*-Si_3_N_4_	193.22	193.74	0.123	140.60	112.87	10.940	1.231
*t*-Si_2_GeN_4_	179.17	178.88	0.081	122.27	98.38	10.827	1.216
*t*-SiGe_2_N_4_	167.22	167.20	0.006	112.57	89.59	11.367	1.283
*t*-Ge_3_N_4_	147.15	146.98	0.058	99.47	74.95	14.058	1.637
*c*-Si_3_N_4_	292.98	292.98	0	269.29	243.56	5.017	0.528
*c*-Si_2_GeN_4_	262.92	262.92	0	236.36	207.84	6.421	0.686
*c*-SiGe_2_N_4_	247.09	247.09	0	208.72	194.06	3.640	0.378
*c*-Ge_3_N_4_	218.24	218.24	0	180.81	164.07	4.854	0.510
*m*-Si_3_N_4_^1^	168.41	162.83	1.685	118.92	101.12	8.089	0.968
*m*-Si_2_GeN_4_^1^	156.16	152.44	1.205	105.18	89.59	8.004	0.905
*m*-SiGe_2_N_4_^1^	176.94	165.26	3.130	93.00	62.06	19.954	2.557
*m*-Ge_3_N_4_^1^	129.33	123.55	2.286	82.36	63.68	12.791	1.513

^1^ Ref. [45].
